# Molecular-level similarity search brings computing to DNA data storage

**DOI:** 10.1038/s41467-021-24991-z

**Published:** 2021-08-06

**Authors:** Callista Bee, Yuan-Jyue Chen, Melissa Queen, David Ward, Xiaomeng Liu, Lee Organick, Georg Seelig, Karin Strauss, Luis Ceze

**Affiliations:** 1 Paul G. Allen School of Computer Science & Engineering, University of Washington, Seattle, WA USA; 2 Microsoft Research, Redmond, WA USA; 3 Department of Electrical & Computer Engineering, University of Washington, Seattle, WA USA

**Keywords:** Machine learning, DNA computing and cryptography, Information technology

## Abstract

As global demand for digital storage capacity grows, storage technologies based on synthetic DNA have emerged as a dense and durable alternative to traditional media. Existing approaches leverage robust error correcting codes and precise molecular mechanisms to reliably retrieve specific files from large databases. Typically, files are retrieved using a pre-specified key, analogous to a filename. However, these approaches lack the ability to perform more complex computations over the stored data, such as similarity search: e.g., finding images that look similar to an image of interest without prior knowledge of their file names. Here we demonstrate a technique for executing similarity search over a DNA-based database of 1.6 million images. Queries are implemented as hybridization probes, and a key step in our approach was to learn an image-to-sequence encoding ensuring that queries preferentially bind to targets representing visually similar images. Experimental results show that our molecular implementation performs comparably to state-of-the-art in silico algorithms for similarity search.

## Abstract (web summary)

Storage technology based on DNA is emerging as an information dense and durable medium. Here the authors use machine learning-based encoding and hybridization probes to execute similarity searches in a DNA database.

## Introduction

Recent advances in DNA nanotechnology have demonstrated synthetic DNA’s ability to carry out molecular computations for biochemical applications, such as gene expression classification^1–4^. These applications reflect a paradigm shift in the field of DNA computing, away from parallel computing in the style of Adleman’s solution to the traveling salesperson problem^5^, and towards DNA strand-displacement circuits^6,7^ and algorithmic self-assembly^8,9^. This shift was motivated by the recognition that encoding combinatorial problems requires synthesizing exponential amounts of DNA, and that synthetic DNA is better suited to implement circuits, which autonomously analyze information already encoded in the concentrations and sequences of nucleic acid molecules. DNA-based digital storage applications provide a unique opportunity to apply parallel computing to large-scale databases already stored in molecular form, without the requirement for exponential amounts of DNA as in Adleman’s work: many database operations are inherently parallelizable, and the amounts of DNA required to perform them grow linearly with database size rather than exponentially. Due to growth in synthesis and sequencing capacity, DNA-based digital storage has become an increasingly attractive technology to address the exponentially widening gap between the volume of media that the world produces and the capacity of traditional storage media (such as solid state, optical, and magnetic storage). Researchers are developing practical systems using synthetic DNA as a storage medium, with several orders of magnitude higher density and durability than current storage technologies^10–16^. To store an arbitrary digital file in DNA, its binary data are translated into a DNA sequence using error-correcting codes that account for limitations and errors in DNA synthesis and sequencing. Because synthetic DNA oligonucleotides are limited in length, the encoded file’s sequence may be split over many hundreds or thousands of oligos, depending on its size. A DNA database may consist of many logically distinct files pooled together, so a required feature of these systems is the ability to retrieve all of the oligonucleotides associated with a single file without having to sequence the entire data pool. This is typically accomplished by associating each file with a unique pair of short, predetermined sequences that are included on each oligo for that file. To retrieve a specific file from an aliquot of a large pool, these sequences can be used as primers for a polymerase-chain reaction (PCR) that selectively amplifies the target file^15,16^.

The sequences of the PCR primers needed to retrieve a specific file are analogous to a filename, in that they must be stored and remembered separately from the data itself. In database terms, this is referred to as key-based retrieval. Although key-based retrieval might be sufficient for a well-maintained library or archive, modern search and recommendation systems do not assume users know the exact key of the document they are looking for, and thus make heavy use of content-based retrieval. For instance, they allow users to search for words, phrases or even topics that occur within documents, or enable them to use an image as a query to retrieve visually similar images.

Executing key-based retrieval in a DNA database leverages DNA hybridization to perform parallel molecular computing: the PCR primers associated with a particular file are programmed to bind with their intended reverse complements, even in the presence of many millions of potentially off-target sequences. Early formulations of DNA databases^17–20^ proposed that hybridization could also be used to search through the content of the documents in the database. However, these approaches require that semantically similar documents are represented by similar sequences, and this is not possible in a DNA database that allows storage of arbitrary digital data. For instance, while a pair of JPEG and PNG files may represent visually similar images, we cannot rely on their binary encoding (or their DNA encoding) to be similar. The same is true for text or other media that may be encoded or compressed in unpredictable ways.

**Fig. 1:**
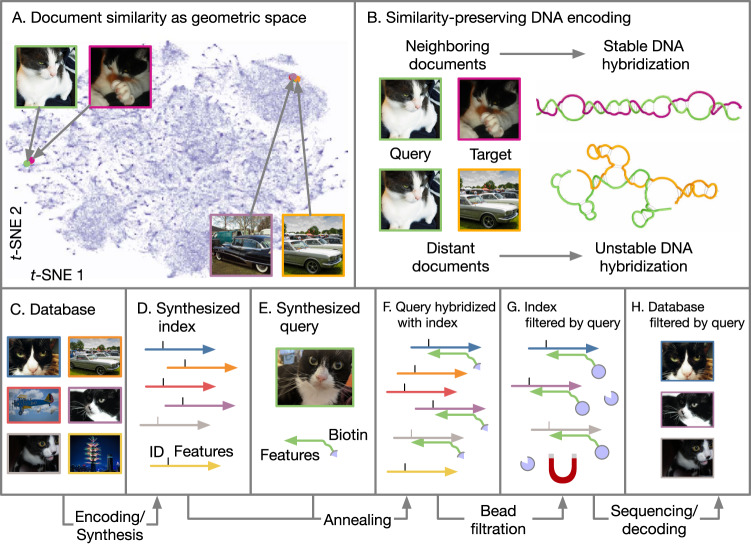
Overview DNA-based similarity search. **A** Illustration of a feature space where neighboring documents are subjectively similar. **B** A similarity-preserving DNA encoding is one where the reverse complement of a query document’s sequence hybridizes with a neighboring target document’s sequence, but not with a distant target’s. Note that the query is color-coded green and the targets with other colors. **C–H** The retrieval process. A database (**C**) is encoded and synthesized to produce an DNA-based index (**D**). Arrowheads indicate 3’ ends of DNA. An encoded query (**E**) is annealed with a sample of the database (**F**), which is filtered with magnetic beads (**G**). The filtered database is sequenced to reveal the IDs of retrieved documents, which are used to look them up in the original database (**H**).

Document similarity is typically formulated as a geometric problem^21^ where each document’s semantic content (rather than its binary representation) is converted to a vector in a high-dimensional feature space, with the property that neighboring feature vectors represent subjectively similar documents. This conversion process is called feature extraction, and there are a variety of methods that work well, depending on the type of document and the goals of the application. For documents like images, intermediate layer activations from neural networks trained on image classification problems (such as VGG16^22^ or AlexNet^23^ tend to perform well as feature vectors for similarity search tasks^24^. Figure 1A illustrates this with a two-dimensional *t*-SNE (t-distributed Stochastic Neighbor Embedding) projection of 4096-dimensional feature vectors extracted using the FC2 layer of VGG16. The images depicted here were not seen by VGG16 during its training; however, because VGG16 was trained on real-world photographs, its effectiveness as a feature extractor generalizes to other real-world images. Database systems facilitate content-based similarity search by creating an index that stores documents’ feature vectors separately from their original data. Searching through the index provides the location of the original document. For instance, a web-based reverse image search engine is an index that allows users to upload an example image, which is used to retrieve the URLs of visually similar images. Index structures do not have to be static: when new data are added into the database, its feature vectors can also be added to the index, without recomputing the feature vectors of other items.

Because similarity search is an efficiently parallelizable problem (each document’s feature vector can be compared with a query simultaneously), it is a good fit for massively parallel DNA computing. An ideal DNA-based index for similarity search encodes feature vectors as DNA sequences such that single-stranded molecules created from an encoded target and the reverse complement of an encoded query are likely to form stable hybridized structures when the query and target feature vectors are neighboring, but not when they are distant (Fig. 1B). Given such an encoding, the index associates each document with a single strand of DNA that contains the document’s ID, alongside with its encoded feature vector (Fig. 1C, D). An encoded query (Fig. 1E) can then be used as a hybridization probe to filter out similar documents from the index (Fig. 1F, G). The filtered index is then sequenced and decoded to recover the IDs of documents that are similar to the query. These documents are then retrieved from a key-based database and displayed to the user (Fig. 1H).

Designing a universal encoding from feature vectors to DNA sequences is difficult because of the high dimensionality of both feature space and DNA sequence space, and because of the nonlinear nature of DNA hybridization. An alternative is to use machine learning to optimize the encoding for a particular dataset. Early researchers^19^ achieved this by clustering the dataset using *k*-means, then mapping each cluster center to a known DNA sequence, which is assigned to each document in that cluster. By reducing content-based retrieval to exact key-based retrieval, their approach sidesteps any issues with unwanted DNA hybridization. However, there is no notion of more or less similar within a cluster: every item in the cluster is retrieved, regardless of its distance to the query. Additionally, once the clusters are chosen, they are static; any additional data added to the database must fit into the existing clusters, even if it would cause the cluster centers to change.

In this work, we greatly expand upon our prior proof-of-principle work^25^ and show how we can scale up computational workflow and molecular image search from tens to over 1.5 million images. We show a path toward overcoming the limitations of fixed clusters by using machine learning techniques to create a continuous feature-to-sequence encoding that preserves similarity. Crucially, the in silico learning step only happens once and the cost of this is amortized over the lifetime of the database. As with the feature extractor, the trained encoder can be applied to new documents not seen during the learning process, provided they share an underlying distribution (e.g., images of the natural world). The trained encoder must translate a new item or a query into its corresponding DNA sequence in silico, but the rest of the computation is carried out molecularly, which accounts for most of the computation for a search query. This allows new items to be freely added or used as queries without retraining, and all of our experiments were performed with documents that were not seen during training.

## Results

### Sequence encoder maps similar images to similar DNA sequences

**Fig. 2:**
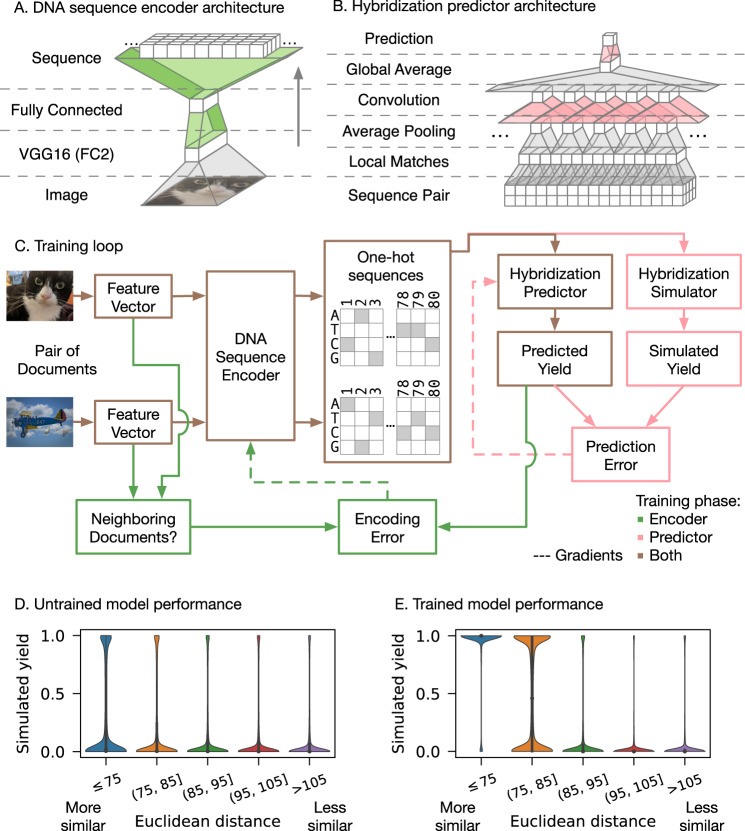
Overview of our neural network architectures and training process. **A, B** The neural network architectures for the image-to-sequence encoder, and the hybridization predictor. Boxes represent layers, and trapezoids represent operations to go from one layer to the next. Only the colored operations have parameters that change during training. **C** The training loop for the neural networks. Lines indicate data flow; dashed lines indicate parameter gradients calculated using backpropagation. Green indicates operations only performed during encoder training, while pink indicates operations used only during yield predictor training. All other operations are used in both training phases. **D** Simulated performance of an untrained model, evaluated on *n* = 1.6 million random pairs of independent images. Each violin depicts the distribution of simulated hybridizations for pairs whose feature vectors’ Euclidean distance lie within a certain range. **E** Simulated performance of a trained model, evaluated on the same set of random pairs (i.e., *n* = 1.6 million random pairs of independent images).

As in our prior work, we focus on encoding feature vectors derived from images, because large datasets and feature extractors are readily available, and similarity between images is easy to visualize. However, our approach can be applied to any type of media, as long as an appropriate feature extractor is available. We use OpenImages^26,27^, a collection of roughly 9 million images, and the FC2 intermediate layer of VGG16^22^, a convolutional neural network designed for image classification, to extract feature vectors. Unlike our prior work, we do not reduce the dimensionality of the VGG16-FC2 vectors prior to encoding. As shown in Fig. 2A, the encoder is a fully connected neural network with one hidden layer that directly translates the feature vectors into softmax-encoded DNA sequences that are 80 nucleotides in length, where each position is represented numerically by a four-channel vector (one channel for each possible base) whose components sum to one. This is a continuous approximation of a one-hot encoding, where one of the four channels would have the value one, and the rest would have the value zero. A continuous approximation is necessary because neural networks must have differentiable operations in order to be efficiently trainable via gradient descent. However, because the softmax encoding is continuous, the encoder may output an indeterminate base for a particular position (for instance, 75% A and 25% G). We do not treat this as a probabilistically random base; to output a sequence from a softmax encoding, we treat it as if it were one-hot and simply take the bases with the maximum values. To encourage the softmax-encoded sequences to have a high maximum value, indeterminate outputs are penalized during training. The goal of the encoder is to map feature vectors to DNA sequences such that a pair of neighboring feature vectors will produce a pair of sequences that are likely to hybridize when one of the sequences is reverse complemented.

### A differentiable hybridization predictor enables efficient optimization of the sequence encoder

In order to optimize the encoder for this goal, we require a way to predict the outcome of a hybridization reaction between one image’s encoded sequence and the reverse complement of another image’s encoded sequence. Predicting the outcome of a hybridization reaction with high accuracy is possible with tools such as NUPACK^28^. However, efficient optimization of neural networks requires that all operations are continuous and differentiable, and NUPACK’s algorithm is neither. In prior work, we used a continuous approximation of the Hamming distance between the two sequences to roughly predict hybridization, but we determined that this would be unlikely to scale to longer sequences. Therefore, a key component of our technique is a differentiable model of DNA hybridization that is trained alongside the encoder (Fig. 2B).

Figure 2C outlines the training procedure, which alternates between encoder and hybridization predictor training phases. In the encoder training phase, a pair of image feature vectors are compared to determine if they are close enough to be deemed “similar”. Supplementary Figure S1 illustrates the relationship between subjective image similarity and feature vector Euclidean distance. Pairs of images with Euclidean distance of 75 or less tend to be consistently similar, so we label these pairs as “similar” and all other pairs as “not similar”. This distance threshold is a property of the feature space, not of any particular image, so it only needs to be chosen once per type of document being encoded. After determining whether or not they are similar, the pair of image feature vectors are encoded independently to produce a pair of softmax-encoded DNA sequences. These sequences are passed to the hybridization predictor, which computes local matches in a small sliding window that allows for misalignments (Supplementary Fig. S3B), then performs pooling and convolution operations to produce a predicted yield. If the predicted yield is low and the documents are similar, or the predicted yield is high and the documents are not similar, the encoder’s parameters are modified (via gradient descent) to correct the error.

During predictor training, the softmax-encoded sequences output by the encoder are discretized into one-hot sequences to ensure that training data are deterministic. The one-hot sequences are passed to the predictor, but they are also output as strings and their reaction is simulated with NUPACK. If the hybridization predictor’s output differs from NUPACK’s output, the predictor’s parameters are modified (via gradient descent) to correct the error. The encoder’s parameters are unchanged during predictor training.

We alternate encoder and predictor training phases until their parameters converge. Because the predictor is much simpler than NUPACK, it will never reach NUPACK’s level of accuracy; however, the model of hybridization it learns is specialized to the encoder’s outputs, and it is good enough that the encoder can learn how to produce pairs of DNA sequences that are likely to hybridize when encoded from neighboring feature vectors (Fig. 2D, E).

### Sequence design enables high-throughput experiments

During training, we withheld a fixed subset of 1.6 million images from OpenImages V4 to be used as our “database” for laboratory experiments. After training our encoder, we transformed each of these images into a DNA sequence using the trained encoder. In addition to the encoded features, each image’s sequence contains a unique, decodable barcode that refers to the ID of the original image, as well as conserved regions to facilitate amplification and processing via PCR (polymerase-chain reaction). Each image’s sequence is short enough to fit on a single synthesizable DNA oligomer (see Supplementary Fig. S4).

Our query images did not come from the OpenImages dataset, and do not exist in the database. To conduct similarity search with an image, we ordered a biotinylated probe oligomer that contains the reverse complement of the query’s encoded feature sequence. We annealed the probe with a sample of the database, and then separated the annealed target/query pairs from the database using streptavidin-conjugated magnetic beads. We then use high-throughput sequencing to reveal which database sequences persist in the separated mixture, and measure how frequently each of them occur.

### After filtering by a query, the most frequently sequenced oligos correspond to images that are visually similar to the query

**Fig. 3:**
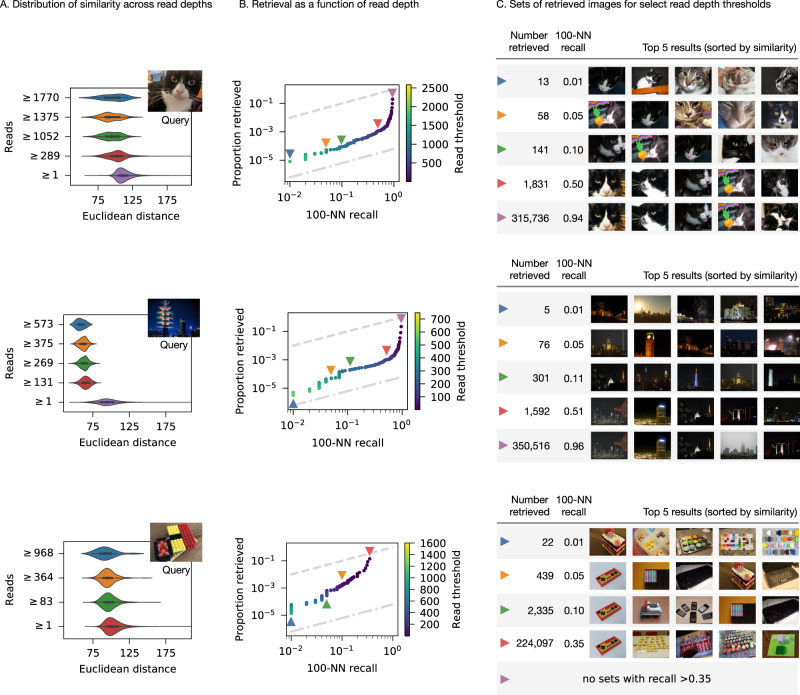
Experimental results for three different query images. Janelle, the cat (top), a building with fireworks (center), and Lego pieces assembled in the shape of sushi (bottom). **A** Distribution of Euclidean distances to the query image, among sets of images with sequencing read depth above a certain threshold. *n* = 1.6 million independent images. **B** The proportion of the entire dataset that must be retrieved (*y*-axis) to retrieve a certain proportion of the 100 most similar images (*x*-axis). Each point represents a threshold for which images with read depth above that threshold are considered “retrieved”. The dashed line indicates chance performance, while the dashed-and-dotted line indicates perfect performance. Colored triangles indicate the thresholds depicted in the other subfigures. **C** The top five closest images to the query from result sets where images above a certain read depth threshold are considered “retrieved”.

Figure 3 shows the experimental results for three different query images. If we consider images with sequencing read counts above a certain threshold to be “retrieved”, we can characterize the set of retrieved images for a variety of thresholds. Figure 3A shows that higher read counts are associated with sets of images that are closer to the query in Euclidean distance. We can quantitatively characterize the quality of a retrieved set by its recall of the 100-nearest neighbors; that is, the number of images in the set that are among the 100 most similar images to the query in the database. Figure 3B shows that, as the read threshold increases, the number of total images in the retrieved set drops very low before you begin to sacrifice nearest-neighbor recall. We can also visually inspect the retrieved set by sorting its contents and displaying the most similar images. Figure 3C shows that, even with very aggressive filtering, the retrieved set still contains images that are relevant to the query. If the read counts for each image are proportional to their concentrations in the filtered mixture, this means that the filtered mixture could be diluted about 1000×, conserving sequencing resources while still retrieving relevant images.

The performance of a similarity search algorithm can be summarized by the curve in Fig. 3B, which measures the proportion of the database that must be retrieved and sorted to achieve a particular 100-nearest-neighbor recall. The dashed line above the curve illustrates a “naive” algorithm that randomly samples the database. To retrieve half of the hundred nearest neighbors, it must retrieve half of the database. The dashed-and-dotted line below the curve illustrates a perfect “oracle” algorithm. To retrieve half of the hundred nearest neighbors, it would retrieve exactly those 50 images from the 1.6 million in the database.

### Performance of molecular filtering is competitive with state-of-the-art electronic algorithms

**Fig. 4:**
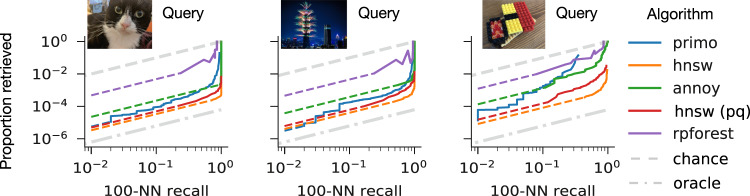
Comparison of our technique (“primo”, shown in blue) with state-of-the-art algorithms for in silico similarity search. Dashed gray and dashed-and-dotted gray lines represent chance performance and perfect performance, respectively. Not all of the algorithms could produce results towards the lower-left (low recall and low proportion retrieved). We assume these algorithms could be stopped early to produce fewer results with a linear decrease in recall; dashed continuations represent these linear interpolations.

Figure 4 places the curve from Fig. 3B in context alongside several state-of-the-art in silico algorithms that were benchmarked using the same query and same database for each of the queries we evaluated experimentally. Implementations of HNSW (hierarchical navigable small world) graphs^29^ are among the top performers on approximate nearest-neighbor benchmarks^30^. HNSW requires building and storing a very large index, which may be difficult to scale to large databases. We also tested a quantized version of HNSW with lower memory utilization, developed by Facebook^31^ (“faiss”, shown in red), annoy^32^ (shown in green), a popular algorithm developed by Spotify, and RPForest^33^ (shown in purple), an algorithm designed for the lowest possible memory utilization. These curves can be interpreted as summarizing the resources (sequencing reads, disk operations, etc.) that must be spent to achieve an acceptable result. On those grounds, our experimental performance is comparable to the state-of-the-art. It is worth noting that executing our molecular technique is still far slower than reading from a disk for databases of this size. However, because the molecular technique is inherently parallel, it may scale to larger databases without requiring significant additional processing time; the same is not true for in silico algorithms that are bottlenecked by the available processing capacity.

### Simulations indicate that further scaling is possible

**Fig. 5:**
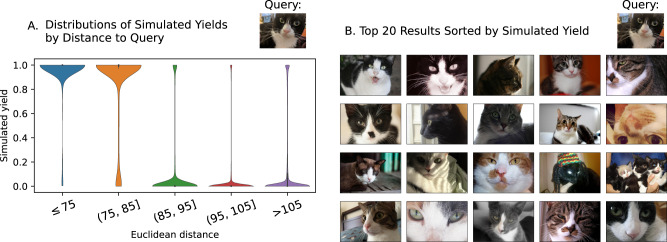
Results of a NUPACK simulation of using a query image of a cat with a larger database consisting of 5.5 million images. **A** Distributions of simulated yields for each target image, categorized by their feature vectors’ Euclidean distances to the query feature vector. The widths of each violin are normalized to be equal. **B** The top 20 images in the database, sorted by the NUPACK-simulated yield of the reaction between their encoded DNA representation and the reverse complement of the query’s DNA representation.

To investigate the effect that increasing the database size might have on search performance, we ran NUPACK simulations on a database of 5.5 million additional images from OpenImages. Figure 5 shows that the highest simulated yields (which should correspond to the most sequencing reads in laboratory experiments) are reserved for images that are visually similar to the query, indicating that aggressive filtering is possible even in larger databases.

## Discussion

Here, we introduced an approach for performing massively parallel molecular image search. In our technique, data are encoded and stored in such a way that the storage substrate, synthetic DNA, also behaves as a computational element by performing DNA hybridization. Computer architects refer to this as in-memory computing or near-data processing, because it avoids the bottleneck of shuttling data between memory and the CPU. It is not a general-purpose computing paradigm, but it is still very powerful because it is capable of efficient parallel computation over high-dimensional data. This basic mechanism can be generalized to broader tasks such as pattern classification and time series analysis^34^.

A limitation to our approach is that the search paradigm (e.g., visual similarity search) is fixed when the database is created, so a user is limited in the way they can search but they do not need to know what to search for. For example, we did not know that there would be images of tuxedo cats in the database before we conducted our search; the query image came from outside of the database. We did, however, know that we were using a query image to search for similar images. Another potential limitation is the long latency (minutes to hours) to complete a single query. However, it is possible to compensate for this and achieve high throughput through batch processing. Furthermore, given a sufficiently large electronic database (e.g., one that does not fit in memory), a single query could require comparably long latency and significant energy consumption.

DNA is an attractive medium for near-data processing because it can perform this kind of computation without a significant expenditure of energy. Training the data-to-DNA encoder is an upfront cost that is only paid once per data type (e.g., natural images). Encoding data as DNA sequences and then synthesizing it as DNA oligonucleotides requires energy, but this energy cost is paid only once per item added to the database. For a single search operation, the query must be encoded and synthesized as well, but the majority of the computation happens when copies of the query molecule hybridize with the database, which releases energy. This is not to say that the computation is “free”: accelerating the hybridization reaction with annealing requires heat, and reading out the results with a DNA sequencer requires energy that is proportional to the number of desired results. However, low-power DNA sequencing techniques such as nanopore sequencing can make this cost negligible. One could imagine a low-power system that carries test tubes of DNA already primed for computation, requiring only mixing or gentle heating of the solution to trigger precipitation of the results.

An additional modification to our system could be changing the size of the feature space. Eighty nucleotides were used in this work due to synthesis length restrictions in place at the time. One could use longer feature regions, but note that hybridization specificity begins to break down at longer sequence lengths because of nonspecific interactions and secondary structure formation. Conversely, feature lengths could be shorter but this would give the encoder fewer degrees of freedom to optimize. There are theoretical frameworks for reasoning about the embeddings of discrete sequence spaces into continuous vector spaces, but not the other way around, so any optimizations of sequence length must be discovered experimentally.

This paper detailed the demonstration of similarity search of digital information in DNA and compared its potential efficiency with electronic systems. The results suggest that, as DNA data storage becomes more practical and scales to larger datasets, similarity search in DNA form is an attractive possibility compared to electronic systems. Combining DNA data storage with similarity search support may offer a path to viable hybrid molecular-electronic computer systems. To integrate DNA data storage and search with existing computing infrastructure, automation is essential. The mechanisms required for automating the protocol, such as magnetic bead extraction, thermocycling, retrieval from stored pools of DNA, input/output with DNA sequencers, and synthesizers, are viable in digital microfluidics systems, e.g., PurpleDrop^35,36^, which offer a path to scalable and low-cost automation.

## Methods

### Feature extraction

To extract image features, we processed each image with VGG16, a convolutional neural network designed for image classification. The weights were loaded from the publicly available trained model and left unchanged during our processing. We used the activations of FC2 (the second fully connected layer) as 4096-dimensional feature vectors.

Supplementary Figure S1 illustrates the relationship between subjective image similarity and feature vector Euclidean distance. Pairs of images with Euclidean distance of 75 or less tend to be consistently similar, so during training we label these pairs as “similar” and all other pairs as “not similar”.

### Sequence encoding

The sequence encoder is a fully connected neural network. Its topology is depicted in Supplementary Fig. S2. The 4096-dimensional FC2 vectors are fed into a 2048-dimensional hidden layer with a rectified linear activation, followed by an output layer with a “one-hot” sequence representation that is 80 nucleotides in length. In this representation, each sequence position has four channels, one for each base. A softmax activation function is applied that forces each position’s channels to sum to 1. A DNA sequence can be read off by picking the channel with the maximum activity at each position.

The one-hot representation can produce indeterminate bases (for example, if all four channels at a position have a value of 0.25). Because of this, a regularization is applied during encoder training to minimize the entropy at each position. This encourages each position to have a well-defined maximum, which improves the accuracy of the yield predictor.

The yield predictor takes a pair of one-hot sequence representations and produces an estimate of the yield of the hybridization reaction between the first sequence and the reverse complement of the second sequence. It is structured as a convolutional neural network (Supplementary Fig. S3A). The network makes use of a local match layer (Supplementary Fig. S3B) that produces vectors of possible matches between each window of 3-mers. This encourages the predictor to make use of any unaligned matches between the two sequences.

### Training

During each round of encoder training, we draw a batch of pairs of feature vectors from the training set where half of the pairs are labeled “similar” (the Euclidean distance between the feature vectors in the pair is 75 or less). The batch of pairs is processed by the encoder, which outputs pairs of one-hot sequences. These are then processed by the yield predictor, which outputs the estimated yield of the hybridization reaction between the first sequence and the reverse complement of the second sequence. The estimated yield of each pair in the batch is used along with the similarity labels (0 for “not similar” and 1 for “similar”) to compute the mean cross-entropy for the batch. We use the cross entropy as loss function because it penalizes similar images with low estimated yield, dissimilar images with high estimated yield, and any estimated yields that are neither high nor low. The parameters of the encoder are modified (via gradient descent) to minimize the mean cross-entropy. The yield predictor’s parameters are not changed during encoder training.

In order to use gradient descent, the one-hot sequence representations cannot be fully discrete. This can create positions with indeterminate bases, which may interfere with the yield predictor. To discretize the one-hot sequences as much as possible, we add an additional regularization term to the encoder to minimize the per-position entropy of the one-hot sequences. This regularization must be gently applied; if it is too strong, the gradients will explode and training will fail.

During each round of yield predictor training, we draw a random batch of pairs of feature vectors (unlike the encoder batches, these are not conditioned to have a particular distribution of similarity). The batch is processed as above to output the estimated reaction yield.

To simulate the reaction yield with NUPACK, the pairs of sequences are discretized. The first sequence in the pair is treated as the target, and a reverse primer is appended as in Fig. S4A. We do not append the forward primer, barcode, or internal primer as these regions will be double stranded during retrieval. The second sequence in the pair is treated as the query, and six bases of the reverse primer are appended, and the sequence is reverse complemented, as in Fig. S4D. The target and query sequences are processed with NUPACK at 21 °C using default DNA parameters and an equal molar concentration of 1 nM for both query and target. The simulated reaction yield is computed by dividing the final concentration of the query-target duplex by the initial concentration of 1 nM.

We compute the cross entropy between NUPACK’s simulated yield and the predictor’s estimated yield for each pair in the batch. The parameters of the yield predictor are modified (via gradient descent) to minimize the mean cross-entropy for the batch. The encoder’s parameters are not changed during predictor training.

### Barcodes

Document IDs are integers in the range 0–16,777,215. Because our database only contains 1.6 million entries, we space out their IDs by mapping them to an ID in the full range using a pseudo-random permutation. To construct a DNA barcode from an ID, the randomized ID is first split into to four six-bit symbols. These are encoded with a Reed-Solomon error-correcting code to produce a codeword with six symbols. Each symbol is converted into a five-nucleotide homopolymer-free DNA subsequence using a codebook with 64 entries. The final 30-nucleotide barcode is the concatenation of these subsequences.

To decode a 30-nucleotide barcode, it is split into six five-nucleotide components, and each step of the code is reversed. Limited substitutions can be corrected, but if the sequence cannot be decoded, or it decodes to an ID that is unused, it is rejected as an invalid barcode.

### Oligo layout

Supplementary Figure S4 depicts the layouts of our synthesized DNA oligomers, as well as the layouts of double-stranded complexes formed during processing. Each document in the database is associated with a single DNA oligomer (Supplementary Fig. S4A) that contains the barcode and feature regions that are unique to that document. In addition to these unique regions, each database oligo contains three conserved regions (denoted FP, RP, and IP) that are the same across all documents. PCR with FP and RP* is used to create additional copies of all database strands (Supplementary Fig. S4B), to prepare for hybridization and sequencing. Linear PCR with IP* is used to create partially double-stranded copies of each database strand that leave the feature region exposed (Supplementary Fig. S4C). Primer sequences are in Supplementary Table 1.

Supplementary Fig. S4D depicts the layout of a query oligo. During retrieval, the reverse complement of the query document’s features are synthesized along with a 5’ biotin, a short spacer, and the reverse complement of first six bases of RP (which serve as a hybridization toehold). The biotinylated query can react with the exposed feature region of any database oligo (Supplementary Fig. S4E). If the resulting complex is sufficiently stable, it can be filtered from the rest of the database using streptavidin-conjugated magnetic beads.

### Benchmarking of in silico algorithms

The in silico similarity search algorithms we compare against in Fig. 4 all perform an Approximate Nearest-Neighbor (ANN) search. Given a dataset, they create an index structure that is traversed using a given query’s feature vector, to retrieve the documents whose feature vectors are nearest to that query. An approximate search does not scan the entire database, but this may cause it to miss some of the nearest neighbors. We define the candidate set as the subset of the database that is scanned (i.e., retrieved from memory and compared to the query). The candidate set is analogous to the set of “retrieved” documents we define by varying the read depth threshold for our lab experiments.

For each algorithm in Fig. 4, and for each of the three queries, we collected the candidate sets for a variety of algorithm-specific parameters that give users control over the specificity of the ANN search. The size of each candidate set (divided by the size of the full database) gives us the proportion retrieved (the *y*-axis of Fig. 4), whereas the number of true nearest neighbors in each candidate set (out of 100) gives us the 100-nearest-neighbor recall (the *x*-axis of Fig. 4).

Some algorithms could not retrieve candidate sets below a certain size for any of the attempted parameters. For these, we assume that uniform subsampling would equally limit both the size of the candidate set and the number of nearest neighbors retrieved. This assumption gives us the dashed colored lines for each algorithm in Fig. 4.

### Reagents

The 1.6 million oligos that make up the database were ordered from Twist Bioscience. Biotinylated probe oligos were ordered from IDT. Streptavidin-coated magnetic beads (Dynabeads MyOne Streptavidin T1) was purchased from Thermo Fisher Scientific. USER enzyme was ordered from New England Lab.

### Laboratory protocol

The general workflow of a similarity search experiment is divided into eight steps (Fig. S5): (1) enrichment of a synthesized oligo pool using PCR, (2) linear amplification of the pool using a forward primer, (3) linear amplification using an internal primer, (4) hybridization experiment using a query strand, (5) magnetic bead extraction, (6) releasing of bead captured strands using digestion of USER enzyme, (7) PCR enrichment of the released oligos, and (8) ligation to Illumina adapters for sequencing.

A DNA pool synthesized from Twist Bioscience was PCR amplified by mixing 1 µL of 1 ng/µL of the pool, 1 µL of 10 µM forward primer, 1 µL of 10 µM reverse primer, 10 µL of 2X KAPA HIFI enzyme mix, and 7 µL of molecular grade water. PCR was performed in a thermocycler with the following protocol (1) 95 °C for 3 min, (2) 98 °C for 20 s, (3) 56 °C for 20 s, (4) 72 °C for 20 s, (5) go to step 2 for about 15 cycles, and (6) 72 °C for 30 s. The amplified product was purified using QIAGEN PCR Purification Kit (Cat No: 28104). The sample concentration was measured using Qubit 3.0 fluorometer.

This enriched Twist pool was mixed with 100 times more of the Forward Primer (e.g., [FP]/[pool] = 100) at 500 nM of the pool. 20 µL of this mixture was mixed with 20 µL of 2X KAPA HIFI enzyme mix, followed by linear amplification with the following protocol: (1) 95 °C for 3 min, (2) 98 °C for 20 s, (3) 62 °C for 20 s, (4) 72 °C for 20 s, (5) go to step 2 for 2 time, and (6) 72 °C for 30 s. The mixture contains 250 nM of double-stranded DNA (dsDNA) and 750 nM of single-stranded DNA (ssDNA).

The sample was linearly amplified again using an Internal Primer (IP) by mixing 40 µL of the 250 nM dsDNA mixture, 12 µL of 10 µM Internal Primer (IP), and 12 µL of 2X KAPA HIFI enzyme mix. Linear amplification was performed with the following protocol: (1) 95 °C for 3 min, (2) 98 °C for 20 s, (3) 56 °C for 20 s, (4) 72 °C for 20 s, and (5) 72 °C for 30 s. The mixture contains 156 nM of fully dsDNA pool and 468 nM of partially dsDNA with feature region exposed (feature region will hybridize to a query strand).

6.4 uL of the mixture (containing 156 nM of the fully dsDNA pool and 468 nM of partially dsDNA) was mixed with 1 uL of a query strand at 10 nM, 10 uL of 2 M sodium chloride buffer, and 2.6 uL molecular grade water. This resulted in a 1:100 ratio of query to the fully dsDNA pool and a final concentration of the fully dsDNA pool at 50 nM. This mixture was annealed in a thermocycler by heating up to 95 °C for 3 min and then slowly cooling down to 21 °C at the rate of 1 °C per 20 min.

Thirty micrograms of Streptavidin-coated magnetic beads (Dynabeads MyOne Streptavidin T1, Thermo Fisher Scientific) was used for 1 pmole of a query strand. The beads were washed three times in binding and washing buffer (5 mM Tris-HCl (pH 7.5), 0.5 mM EDTA, 1 M NaCl), then added to the hybridization sample at room temperature. After incubating at room temperature for 15 min, the samples sat on a magnet rack to recover the beads and binding DNA. The supernatants were removed, and the beads were washed three times using 100 µL of binding and washing buffer. The beads binding DNA was resuspended in 50 µL 1× elution buffer containing 10 mM tris-Cl, at pH 8.5. The resuspended samples were digested using USER enzyme by mixing 50 µL of the sample with 2 µL of USER enzyme, and 5.8 µL of NEB 10× cut smart buffer at 37 °C for 20 min. The sample sat on a magnetic rack for 1 min, and the supernatants was recovered.

Two microliters of the recovered solution from the previous step was mixed with 1 uL of 10 uM forward primer, 1 µL of 10 µM reverse primer (RP), 10 µL of 2X KAPA HIFI enzyme mix, and 6 µL of molecular grade water for PCR. PCR was performed in a thermocycler using the following protocol (1) 95 °C for 3 min, (2) 98 °C for 20 s, (3) 62 °C for 20 s, (4) 72 °C for 20 s, (5) go to step 2 for a varying number of times depending on the recovery yield of beads extraction, and (6) 72 °C for 30 s. 2 µL of the amplified sample was mixed with 1 µL of 10 µM forward primer with an overhang of a randomized region (25 N), 1 µL of 10 µM reverse primer (RP), 10 µL of 2X KAPA HIFI enzyme mix, and 6 µL of molecular grade, followed by the following thermocycling protocol: (1) 95 °C for 3 min, (2) 98 °C for 20 s, (3) 62 °C for 20 s, (4) 72 °C for 20 s, (5) go to step 2 for a varying number of times depending on the recovery yield. This PCR step added a randomized region to the sample for the diversity need of Illumina NextSeq. The size of the PCR product was verified using QIAGEN bioanalyzer. The amplified product was ligated to Illumina sequencing adapters with TruSeq Nano reagents and protocol. The ligated samples were sequenced using Illumina NextSeq.

### Reporting summary

Further information on research design is available in the Nature Research Reporting Summary linked to this article.

## Supplementary information

Supplemental Information

Reporting Summary

## Data Availability

Target images are publicly available via Google: https://storage.googleapis.com/openimages/web/download_v4.html. VGG16 feature vectors can be extracted from images using a publicly available model (via Keras): https://keras.io/api/applications/vgg/#vgg16-function. Encoded DNA sequences, query images, sequencing data, and analysis generated in this study have been deposited in the Github database:https://github.com/uwmisl/primo-similarity-search (10.5281/zenodo.5090717)^37^. With the exception of the query images, all images were collected from Open Images V4, a dataset of over 9 million URLs for images with Creative Commons licenses. Of these, ~1.7 million are hosted by the CVDF and available for download; the rest are raw Flickr URLs and may or may not be available. For the image database used in our experiments, we took 1.6 million images from the hosted set. For training, we took images from the full set of 9 million that were not used for training, testing, or experiments.

## Code availability

The code is available here: https://github.com/uwmisl/primo-similarity-search (10.5281/zenodo.5090717)^37^.
