# Supplementary material for: Molecular-level similarity search brings computing to DNA data storage
Source: Nat Commun. 2021 Aug 6;12:4764. doi: 10.1038/s41467-021-24991-z (PMC8346626; doi:10.1038/s41467-021-24991-z)
Supplement: Supplementary file 1 — Supplemental Information [file 41467_2021_24991_MOESM1_ESM.docx]

Supplementary Information for

Molecular-level Similarity Brings Computing to DNA Data Storage

Callista Bee, Yuan-Jyue Chen, Melissa Queen, David Ward, Xiaomeng Liu, Georg Seelig, Karin Strauss, Luis Ceze.

Correspondence to: kstrauss@microsoft.com or luisceze@cs.washington.edu.

**This PDF file includes:**

Supplementary Figures. S1 to S5


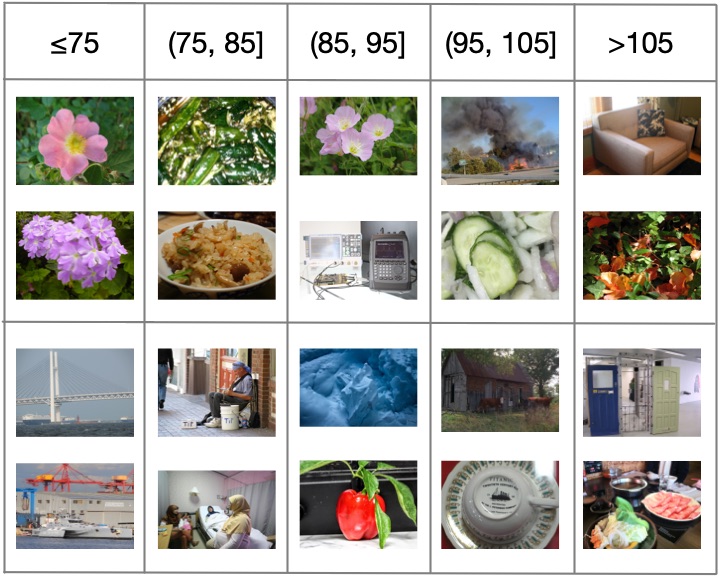


Supplementary Figure S1.

Illustration of the relationship between pairwise feature-vector Euclidean distance and pairwise subjective similarity. Each column represents a range of Euclidean distances, and each row depicts a pair of images where their feature-vector Euclidean distance falls in that range.


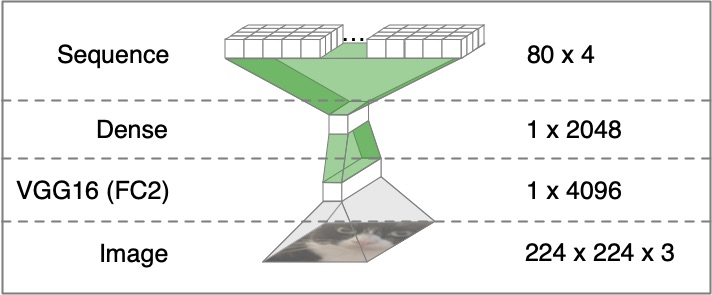


Supplementary Figure S2.

Structure of the sequence encoder network. Layers are opaque, and transformations are translucent. The dimensionality of each layer is shown on the right. Only the transformations highlighted in green have parameters that change during training.


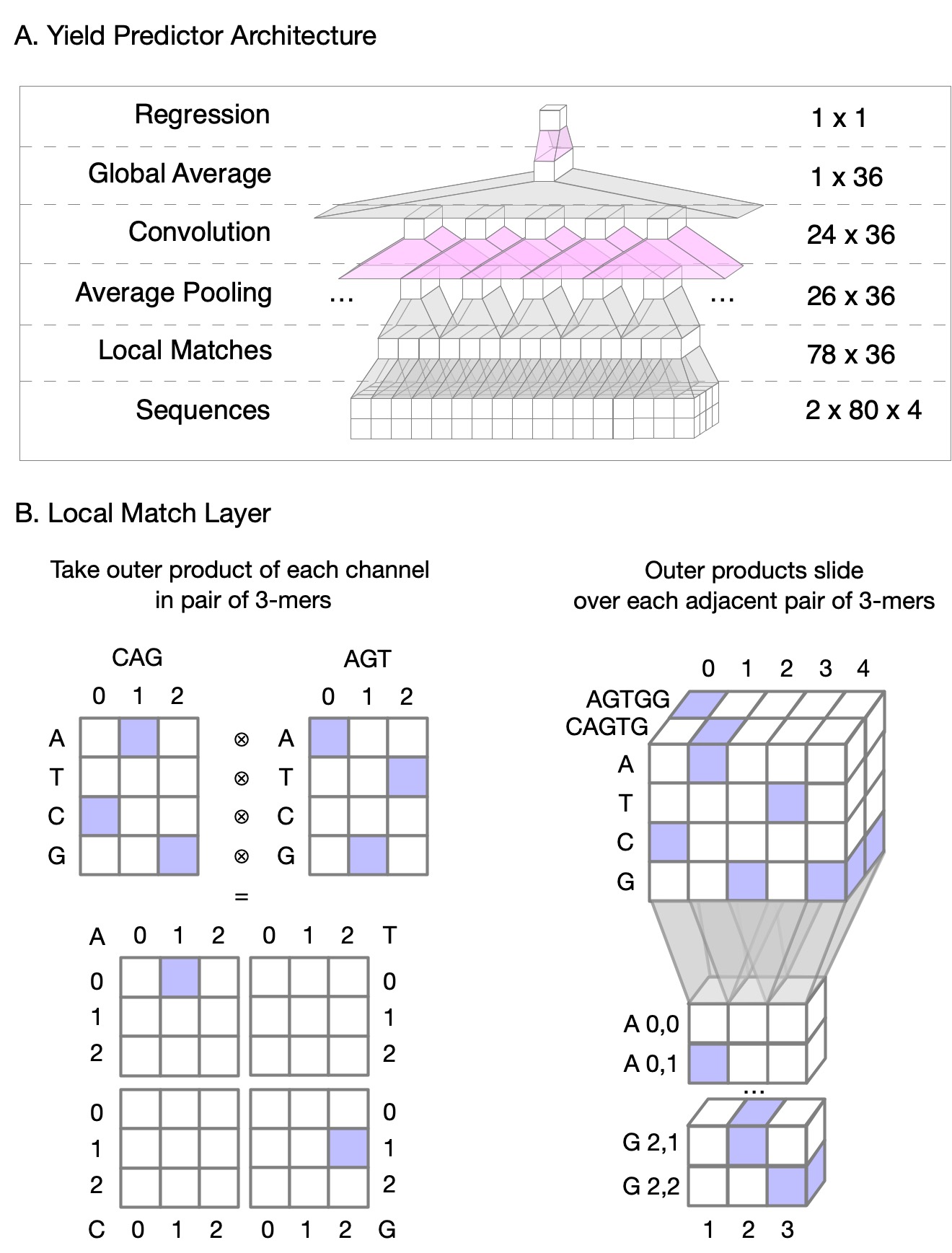


Supplementary Figure S3.

(A) Structure of the yield predictor network. Only the transformations highlighted in pink have parameters that change during training. (B) Illustration of the “local match” operation. Blue cells have a value of 1, and white cells have a value of 0.


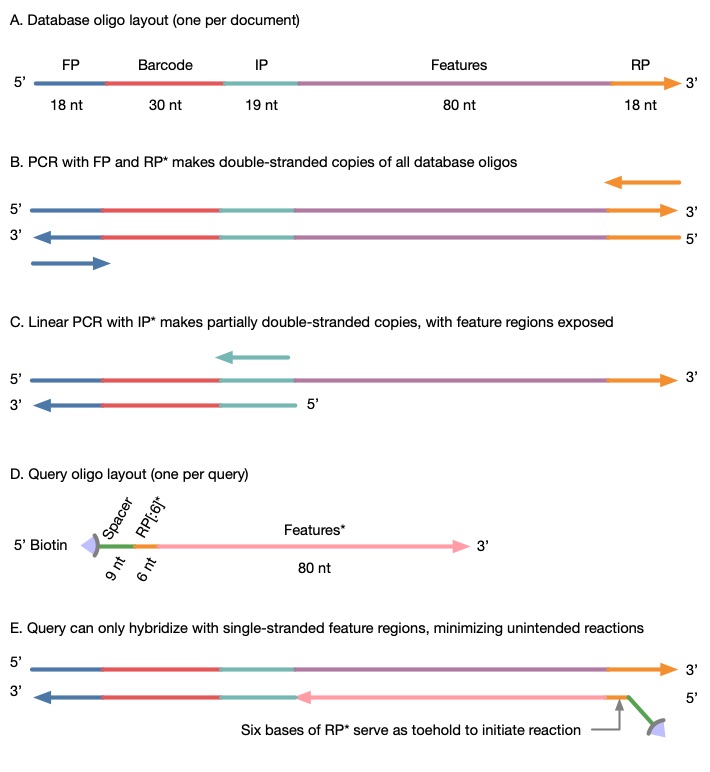


Supplementary Figure S4.

Layouts of single-stranded oligomers and intended double-stranded complexes. Arrowheads indicate 3’ ends of DNA. Asterisks (*) indicate the reverse complement of a DNA sequence.


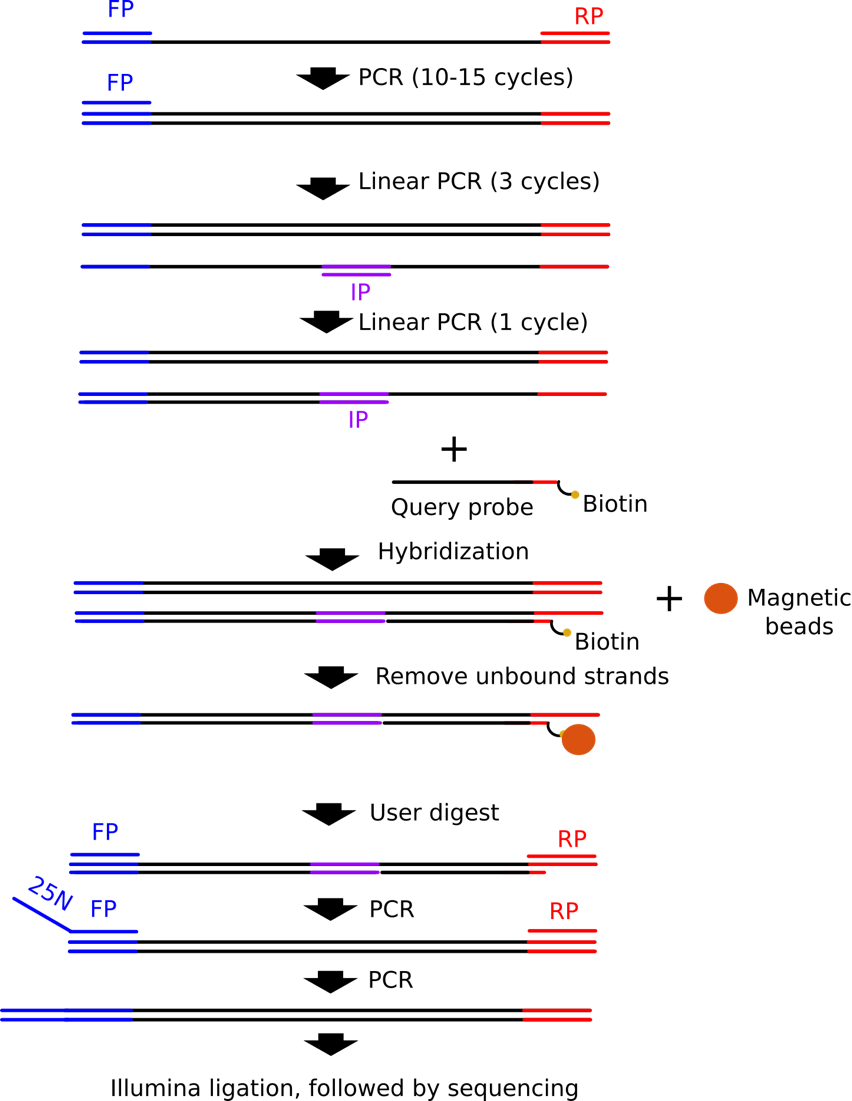


**Supplementary Figure S5.** Workflow of a similarity search experiment. A large DNA pool is PCR amplified using a forward primer (FP) and a reverse primer (RP). The enriched product is linearly amplified using the forward primer for 3 cycles. The sample is linearly amplified using an internal primer (IP) to make partially double-stranded copies, with feature region exposed. This mixture is then hybridized with a query strand, followed by magnetic bead extraction. The extracted strands are released from the beads using USER enzyme digestion. The released sample is PCR enriched using FP and RP. The sample is PCR again using RP and FP with a 25N overhang to create a randomized region for the diversity need of Ilumina NextSeq. The sample is ligated to Illumina adapter, followed by next-generation-sequencing.

| Name | Sequences | Length (bp) |
| --- | --- | --- |
| FP | GCCGACCAGTTTCCATAG | 18 |
| IP* | CGGACAAATACTGAGTGCT | 19 |
| RP* | CAGGAGGTTGTTGAGGAC | 18 |

**Supplementary Table 1. Primer sequences.**
